# Use of noninvasive ‘bug-eggs’ to enable comparative inferences on genetic mating system with and without parental information: A study in a cattle egret colony

**DOI:** 10.1371/journal.pone.0183153

**Published:** 2017-08-30

**Authors:** Carolina Isabel Miño, Elaine Dantas de Souza, Emmanuel Moralez-Silva, Talita Alvarenga Valdes, Vera Lúcia Cortiço Corrêa Rodrigues, Sílvia Nassif Del Lama

**Affiliations:** 1 Instituto de Biología Subtropical (IBS), Nodo Iguazú, Universidad Nacional de Misiones (UNaM)–CONICET, Puerto Iguazú, Misiones, Argentina; 2 Departamento de Genética e Evolução, Universidade Federal de São Carlos, São Carlos-SP, Brazil; 3 Superintendência de Controle de Endemias, Mogi-Guaçu, SP, Brazil; University of Padova, ITALY

## Abstract

Colonial waterbirds such as herons, egrets and spoonbills exhibit ecological characteristics that could have promoted the evolution of conspecific brood parasitism and extra-pair copulation. However, an adequate characterization of the genetic mating systems of this avian group has been hindered by the lack of samples of elusive candidate parents which precluded conducting conventional parentage allocation tests. Here, we investigate the genetic mating system of the invasive cattle egret using hematophagous insects contained in fake eggs to collect blood from incubating adults in a wild breeding colony. We tested a protocol with a previously unused Neotropical Triatominae, *Panstrongylus megistus*, obtained blood samples from males and females in 31 nests built on trees, drew blood from 89 nestlings at those nests, and genotyped all samples at 14 microsatellite *loci*, including six new species-specific *loci*. We comparatively addressed the performance of parentage allocation *versus* kinship classification of nestlings to infer the genetic mating system of cattle egrets. In line with previous behavioral observations, we found evidence in support of a non-monogamous genetic mating system, including extra-pair paternity (EPP) and conspecific brood parasitism (CBP). Parentage allocation tests detected a higher percentage of nests with alternative reproductive tactics (EPP: 61.7%; CBP: 64.5%) than the kinship classification method (EPP: 50.0%; CBP: 43.3%). Overall, these results indicate that rates of alternative reproductive tactics inferred in the absence of parental genetic information could be underestimated and should be interpreted with caution. This study highlights the importance of incorporating samples from candidate parents to adequately determine the genetic mating system of a species. We expand knowledge on the reproductive tactics of colonial waterbirds, contributing novel data on the genetic mating system of the cattle egret, valuable for the design of management strategies for this invasive bird.

## Introduction

Mating systems, i.e., the way in which females and males organize during breeding seasons, are dynamic and can be remarkably variable among species and populations, as well as within populations [1]. Mating systems influence many aspects of the evolutionary ecology of populations, and are, in turn, affected by factors such as the adult sex ratio, type and duration of pair bonds, (un)synchronized fertility, the form and degree of parental care, territoriality, and breeding density, among others [1]. In many species, however, the social (observed) mating system does not always comply with the genetic mating system [2, 3]. Hence, the adequate characterization of the actual mating system of a species requires that genetic studies are carried out as a complement to field observations on social reproductive behavior. For almost three decades, molecular tools have been extensively adopted by field researchers to study mating systems, reproductive strategies and sexual selection [4]. The genetic mating system of a species is usually inferred by conducting conventional DNA-based parentage allocation tests, based on the comparison of the genotypes of attending adults and those of the progeny [5]. Such methods have revealed that alternative reproductive strategies, such as extra-pair copulations (EPC) and conspecific brood parasitism (CBP)–when a female lays eggs in the nests of another female–are fairly common in passerines and non-passerines [2,6,7].

Colonial breeder birds, such as egrets, herons and spoonbills (Order Ciconiiformes), exhibit characteristics that could have promoted the evolution of alternative reproductive tactics and are, thus, expected to have rather high rates of extra-pair paternity (EPP) and CBP [6–9]. For example, dense colonies, floater fertile females present in colonies, and EPCs may set the stage for the occurrence of both behaviors in such species [6–9]. In this avian group, making detailed focal observations on the behavior of wild reproductive adults is difficult, because, in the colonies, many reproductive pairs congregate on rather small spatial scales. Even if observations could be made, extrapolating the social behavior to the genetic mating system may lead to inadequate inferences [2]. Therefore, the application of DNA-based techniques would be the most direct approach to addressing the genetic mating system of colonial waterbirds. However, this avian group poses another challenge for this type of approach: attending adults are not easily captured at nests and genetic samples of complete families are therefore not available for conducting conventional parentage analyses [10, 11]. Thus, so far, most inferences on the reproductive behavior of adults in wild waterbirds have been made mainly applying a methodological approach based on DNA-based kinship classification of nestling-pairs within nests, in the absence of parental genetic information [2, 11–16]. However, there is ample agreement that greater statistical power is achieved in kinship analyses when more family members are sampled [5, 17]. In contrast, identifying potential parents from a sample of offspring when neither parent is known is more challenging [18]. Complete familial sampling should therefore provide more accurate inferences on the genetic mating system of a species. Yet, to the extent of our knowledge, no study has specifically addressed the performance of both approaches to infer genetic mating systems using the same genotypic dataset.

An appealing sampling strategy for studying species with elusive reproductive adults is the use of blood-sucking insects (Triatominae, Reduviidae, Heteroptera, Insecta) [19–21] to collect blood samples. This bleeding technique has been described in detail by von Helversen et al (1986) [19] and further developed by Voigt et al. (2004) [20]. Hematophagous bugs are easy to handle and transport to the field, they have a small proboscis and can perforate micro-vessels, releasing anticoagulants into the blood after ingestion, minimizing pain, bruising and allergic reactions, all of which are advantages for blood sampling of animals [21]. The use of Triatominae insects contained in fake eggs, known as the ‘bug-egg’ method, has been developed a decade ago to collect blood from incubating ground-nesting common terns (*Sterna hirundo*) for a variety of metabolic studies [22–24]. This method was also applied to obtain samples from incubating common swifts (*Apus apus*) [25], and, more recently, from medium-sized raptors, such as Montagu’s harriers (*Circus pygargus*) [26] and Eurasian kestrels (*Falco tinnunculus*) [27]. Yet, to the extent of our knowledge, the ‘bug-egg’ method has neither been applied to study species outside those mentioned above nor to study birds in the Neotropical region. Thus, the benefits of this method for collecting blood samples from wild adults remain unexplored in colonially breeding species.

In this study, we investigated the genetic mating system of the cattle egret *Bubulcus ibis* Linnaeus 1758 (Ardeidae, Ciconiiformes) in a natural breeding colony. The cattle egret is an invasive generalist with an extraordinary capacity for dispersion and colonization [28–30]. The invasive potential of cattle egrets may be enhanced by characteristics common to other invasive organisms, such as few predators, a diverse diet, a fast reproductive cycle, high reproductive success with up to two broods per year, and a high recruitment rate. The cattle egret has expanded its range from the southern Iberian Peninsula and sub-Saharan central-eastern Africa, having invaded and successfully established in all continents, except Antarctica [28]. The species can now be found in many different environments, from sea level to over 4000 m.a.s.l. [28]. Like other invasive species, the cattle egret negatively impact native species [31, 32]. In Brazil (South America), for example, it competes for nesting and foraging sites, interacts agonistically with and preys upon the eggs and chicks of endangered birds [33–35].

The mating system of an invasive species can influence the level of genetic diversity of the colonizing group, affecting establishment success and range expansion probability [36, 37]. Pioneering focal observations carried out in natural breeding colonies reported cattle egrets as predominantly monogamous, but also identified EPCs, bigamous and polygamous bonding [38–43]. Supernumerary clutches of up to seven eggs were also detected in natural colonies of this species and interpreted as evidence of CBP [28]. However, intensive mate-guarding during the fertile period of the females would minimize the occurrence of successful EPCs [38]. Thus, the extent to which EPCs lead to fertilizations and extra-pair paternity (EPP) in cattle egrets remains unknown and whether polygamous reproductive behavior corresponds to a non-monogamous genetic mating system is still unclear. Our objectives were to: 1) demonstrate the usefulness of the ‘bug-egg’ method for obtaining blood samples from incubating cattle egrets in nature; 2) test a field protocol with a previously unused Neotropical species of Triatominae; 3) isolate DNA from bug-collected blood and successfully amplified microsatellite markers; 4) conduct conventional DNA-based parentage analyses using the genotypes of complete familial samples (attending male, attending female and nestlings) to infer the genetic mating system; 5) apply the multiple methods approach [11] for kinship classification of nestlings to infer the genetic mating system; and 6) perform a comparative analysis of inferences made in the presence and absence of parental information to test the hypothesis that inferences are more accurate when samples of candidate parents are available. Given previous behavioral evidence supporting alternative reproductive tactics in this species [38–43], we expect to find half-sibs (resulting from EPP), full-sibs (resulting from monogamy), and unrelated nestlings (products of CBP) within broods. Moreover, given that the multiple methods approach is supposedly conservative [11], we expect to find a lower percentage of alternative reproductive tactics with this method than with conventional parentage allocation. Given that distinguishing between half-sibs and unrelated nestlings is more difficult than distinguishing between non-adjacent relatedness categories [44], we also expect that the relative proportions of EPP and CBP inferred in the presence of parental information will differ from those inferred in the absence of such data. We contribute novel data on the actual genetic mating system of this invasive bird species and put forth important remarks regarding inferences on the genetic mating system made in the absence of parental genetic information.

## Material and methods

### Ethics statement

This study was carried out in strict accordance with Brazilian laws for research on wild birds. Permits to handle and rear the insects were obtained from the Brazilian *Superintendência de Contole de Endemias*–SUCEN [CONCEA Process no. 01200.003280/2014-28(355), CIAEP: 01.0347.2014]. Blood samples were collected under a specific permit from the *Instituto Chico Mendes de Conservação da Biodiversidade*–ICMBio (permit No. 20295–2). All methods used related to capturing and handling the birds, banding and blood collection comply with the ICMBio guidelines. According to SUCEN institutional protocols, nymphs raised in the laboratory and used in the field cannot be reincorporated into laboratory populations. Hence, the insects used for blood sampling in the field, regardless of whether or not they had fed on cattle egrets, were anesthetized with diethyl ether inhalation and immediately incinerated. The field studies did not involve endangered or protected species.

### Blood sampling

**Fig 1:**
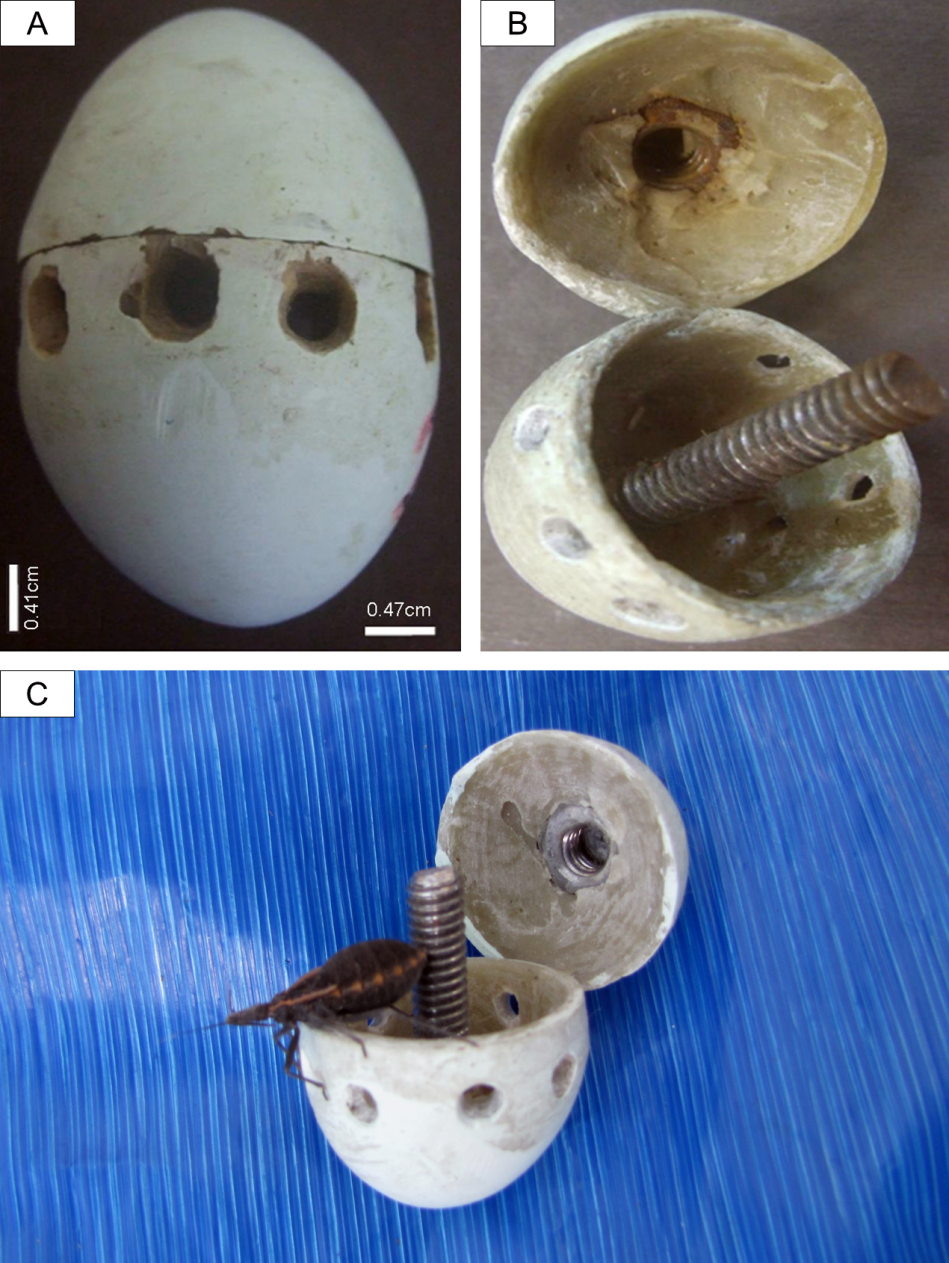
Fake eggs and hematophagous insects used for blood collection of incubating cattle egret adults. Photograph showing: (A) Side view of fiberglass egg mimicking cattle egret egg (approximate dimensions: 45 mm length, 33 mm width) with holes through which the insect projects its proboscis; (B) Interior view of two halves of egg, which are attached with nut and bolt; (C) Fifth instar *Panstrongylus megistus* larva being removed from a fake-egg after having fed on an incubating cattle egret. Note the insect's engorged abdomen.

Sampling was conducted between October and December 2011 in a cattle egret breeding colony established in the city of Rio Claro, state of São Paulo, Brazil (22°30’29”S, 47°35’37”W). During the sampling period, the mean temperature was 22.3°C and mean humidity was 68.3% (data from the *Estação Meteorológica do Centro de Análise e Planejamento Ambiental*–CEAPLA/IGCE/UNESP, Brazil). Throughout the reproductive cycle, the colony had ca. 800 active nests of cattle egrets built in trees between one and three meters in height. As breeding adults often fly away when humans approach (< 30 m distance) [45], blood was obtained from incubating adults employing the ‘bug-egg’ method with a previously unused species of Triatominae. We used fourth and fifth stage flightless larvae of *Panstrongylus megistus* Burmeister 1835 (Insecta: Reduviidae) from a disease-free population reared in a controlled environment, previously starved for fifteen days. This species of insect was chosen because it preferably feeds on birds and also because laboratory-bred nymphs are readily available in several places of the Neotropical region. Fiberglass fake eggs [22] were manufactured to resemble cattle egret eggs in color and size (45 mm length, 33 mm width) (Fig 1A); holes measuring 5 mm Ø spaced at 5-mm intervals were made along the circumference of eggs so that the bugs could project their proboscis out without escaping. One insect was placed inside each fake egg immediately prior to use, and the two halves of the egg were carefully closed with a bolt and nut (Fig 1B), without harming the bugs. Once in the field, ‘bug-eggs’ were placed inside cattle egret nests together with real clutches and tied to the nest with nylon thread. As cattle egrets have biparental incubation and do not have sexual dimorphism, three to six 20-min trials (each with a new ‘bug-egg’) were conducted per nest throughout the day (early morning, midday, afternoon, late afternoon) to increase the odds of sampling both sexes. During the pilot testing of the field protocol, 30% of nests were continuously observed with binoculars while the ‘bug-eggs’ were inside the nests. When the researcher approached the nest to place a ‘bug-egg’ inside, the adult that was incubating the eggs left. Immediately after the researcher moved away from the nest, only one bird returned to incubate the eggs and remained sitting in the nest. After 20 min, the bug-eggs were removed for checking if they had fed on birds’ blood. If the insects had fed, then, cattle egrets’ blood was drawn by perforating the engorged abdomen of the bugs (Fig 1C) with sterilized 29-mm gauge needles coupled to 1-ml syringes previously rinsed in 0.3% EDTA. Blood was also sampled from flightless nestlings aged one week (*n* = 89) that were inside the same nests from which we collected the blood of incubating adults. Blood was obtained from the aorta vein of nestlings using 29-mm gauge needles coupled to 1-ml syringes previously rinsed in 0.3% EDTA. We used EDTA in both sampling procedures in order to impose similar conditions to the blood taken from adults (bug-eggs) and nestlings (vein-puncture). All blood samples were stored in 1.5-ml tubes containing 100% ethanol and kept at -20°C until processed.

### DNA extraction, molecular sexing and microsatellites’ genotyping

Genomic DNA was isolated from blood samples using a phenol:chloroform method followed by ethanol precipitation [46], quantified using a NanoVue spectrophotometer (GE Healthcare, Little Chalfont, UK) and diluted to 20 ng/μl. Samples were sexed by polymerase chain reaction (PCR) amplification of *CHD* genes using P2/P8 primers following the method described by Griffiths et al. [47]. Sex was identified by running PCR products in 9% polyacrylamide gels in vertical electrophoresis. Samples were genotyped at fourteen microsatellites: thirteen species-specific *loci* (seven from [48], plus six new *loci* developed for this study [Table A in S1 File]), and a *locus* isolated in the great blue heron, *Ardea herodias* [49]. PCR protocols and cycling conditions are given in Appendix A in S1 File. A negative control using DNA extracted from six starved *Panstrongylus megistus* larvae was included as a template in all microsatellite amplifications. PCR products were run in a MegaBACE 1000 automatic sequencer (GE Healthcare Life Sciences, Piscataway, NJ, USA) with ET550-R as the internal size standard and alleles were called using Fragment Profiler Software Suite v1.2 (GE Healthcare Life Sciences, Piscataway, NJ, USA). PCRs and allele scoring were repeated at least twice for all samples.

### Genetic diversity and power for parentage and relatedness analyses

Evidence of allele dropout, *stuttering* or null alleles were verified using Micro-checker v2.2.3 [50]. Genotyping error rates, per *locus* and multilocus, were calculated manually for samples that were genotyped in duplicate, based on Pompanon et al. [51]. Deviations from Hardy-Weinberg Equilibrium and Linkage Disequilibrium were tested using Genepop v4.3 [52, 53]. The number of alleles per *locus*, observed heterozygosity, unbiased expected heterozygosity and the Fixation Index, as well as probabilities of exclusion (*PE*) and of identity between siblings (*PI*_*Sibs*_) [54] were computed in GenAlEx v6.5 [55] for a reference sample of 62 presumably unrelated adults of both sexes (social pairs sampled in 31 nests [see below]).

To estimate the power of the set of microsatellites to discriminate full-siblings from half-siblings, as well as full-siblings and half-siblings from unrelated dyads, the Power for Relationship Inference (*PWR*) was computed in Kininfor v.2 [56]. We ran 10^3^ simulated pairs of genotypes for each relationship category, using the empirical marker error rates and allele frequencies computed for the sample of 62 unrelated adults as input data, and set a 5% significance level. The same program was run to compute the multilocus Informativeness Index for Relatedness Inference (*Ir*) and the Informativeness Index for discrimination between two competing relationships (*I*_*R*_), as well as to identify the most suitable of seven different relatedness estimators for the cattle egret genotypic dataset. For these analyses, we set a null hypothesis of parent-offspring versus an alternative hypothesis of full siblings because this provides the highest stringency for inferring relatedness [56], and a 5% significance level.

### Parental allocation analyses

Using the ‘bug-collected’ genotypic information for attending adults, we first checked parent-offspring dyads carrying out conventional DNA-based parentage allocation tests implemented in Cervus v3.0.7 [57]. As an overall goal, we were interested in minimizing the chance of erroneously inferring EPP and/or CBP, which would be the case if a true parent-offspring pair was not identified as such at a given alpha threshold (i.e., type II errors) [58]. Then, to establish the critical LOD score that would provide an average 95% confidence level that the social father was not the true father, we first ran a ‘simulated parentage analysis [58]. Parameters for this analysis included 32 candidate fathers, 0.99 of *loci* typed and 3% overall genotyping error rate. The proportion of candidate fathers sampled was set to 0.73 which is the complement to the conservative initial estimate of EPP in the dataset, which was obtained by counting double *loci* mismatches between social males and offspring (24 out of 89 offspring = 0.27) [58]. To estimate the rate of CBP, we first ran a maternity simulation in Cervus as previously described, but setting the proportion of sampled mothers to 0.90 (complement of 0.10 based upon a double *loci* mismatch in three out of 89 offspring as an initial estimate of CBP). We then used critical LOD scores derived from simulations to carry out ‘paternity’ and ‘maternity’ analyses in Cervus and manually compared the LODs obtained in such analyses to determine which social parents were not related to offspring.

### Relatedness and kinship classification without parental information

We applied the multiple methods approach [11] to classify dyads into relatedness categories (i.e., unrelated, half siblings or full siblings). Briefly, for each nestling-pair within the nests, using Coancestry v1.0.1.7 [59], we computed the pairwise values of the best relatedness estimator for our set of markers and genotypes (identified as described above). Then, using ML-Relate [60] with 10,000 randomly simulated genotypes, we generated maximum likelihood (ML) relatedness hypotheses, testing their significance by computing their probability *versus* the probability of full siblings, which is the relationship expected *a priori* under the assumption of genetic monogamy. In addition, using Colony v2.0.6.2 [61], we partitioned all genotypes of nestlings into family groups (sibship reconstruction). To this end, we ran two set of tests: one accounting for EPP, assuming ‘monogamy for females’ and ‘polygamy for males’, with a 0.73 proportion of candidate fathers sampled (from the Cervus exploratory analyses described above) and a second set of tests accounting for CBP, assuming ‘polygamy’ for both sexes, with a 0.97 proportion of candidate mothers sampled (from the Cervus exploratory analyses described above). The remaining parameters were set equally for both runs: known allele frequencies (computed from 62 unrelated adults, as described above), long runs, full-likelihood method, no updating of allele frequencies and no sibship size prior. Finally, we clustered genotypes into sib-groups applying the Markov Chain Monte Carlo (MCMC) algorithm described by Herbinger [62] implemented in Pedigree v 2.0 (http://herbinger.biology.dal.ca:5080/Pedigree). The final classification of nestling dyads into relatedness categories was determined only if the results of all methods were in agreement; otherwise, dyads were not classified [11].

## Results

### Success of ‘bug-egg’ sampling method

*Panstrongylus megistus* contained in fake fiberglass eggs fed successfully on the blood of cattle egrets under natural conditions (Fig 1C). One hundred twenty blood samples were collected from incubating adults (mean: 4.1 samples per nest) among the 178 trials, representing a 67.4% success rate. Only samples of one sex were obtained in each trial, as indicated by a standard pattern of male or female amplicon bands in the sexing protocol, and also by direct observation of the nests during sample collection. The insects did not feed in 31.4% of trials. In one nest, the incubating adults removed the egg from the clutch twice, representing a rejection rate of 0.01% of trials. After six attempts at the nest in which rejection occurred, four blood samples were obtained. The ‘bug-egg’ method enabled collecting samples from both females and males attending 31 nests (*n* = 62). Bug-collected blood (30–80 μl *per* sample) yielded an average of 407 ng/μl of adult genomic DNA.

### Genetic diversity and power for parentage and relatedness analyses

All adults (using DNA isolated from bug-collected blood) and nestlings (*n* = 89) were genotyped at the 14 *loci*. There was no evidence of large allele dropouts, null alleles and/or stuttering. We found a minimum of two and a maximum of ten alleles per *locus* in the sample of 62 unrelated adults (Table 1, Table B in S1 File). There was no evidence of deviation from Linkage Disequilibrium between any pair of *loci*. *Locus* Bi26, with three alleles, deviated significantly from Hardy-Weinberg Equilibrium (*P* < 0.001) (Table B in S1 File) and had a positive *F*_IS_ value, which could indicate excess homozygotes. *Locus* Bi15 had only two alleles in similar frequencies, and *Locus* Bi28 also showed two alleles with one almost fixed (TableB in S1 File). Thus, to avoid introducing unnecessary sources of error as the loci Bi15, Bi26 and Bi28 were uninformative for relatedness and parentage analyses [63] they were excluded from the genotypic dataset. The final 11-*locus* dataset had higher power and informativeness indexes (*Ir* = 0.79, *I*_*R*_ = 0.86) than the initial 14-*locus* dataset, which further validated the exclusion of the three *loci*. The 11-*locus* dataset had a multilocus error rate of 0.03, a probability of exclusion over 0.99 and a probability of identity between siblings of 0.00064 (Table 1). The mean *Q&R* relatedness value among adults was -0.01 ± 0.00 (95% CI), indicating that they were, on average, unrelated. Simulation results showed that only five *loci* were sufficient to attribute parentage (distinguish PO dyads) with > 0.90 confidence probability (Fig A in S1 File). In addition, simulations results showed that, when using the kinship classification method alone, as few as five *loci* allowed distinguishing full-siblings from unrelated nestlings with a power of > 0.99 (Fig A in S1 File). On the other hand, the power of the 11 *loci* was <0.50 to distinguish full-siblings from half-siblings, and about 0.40 to distinguish half-siblings from unrelated dyads (Fig A in S1 File). Simulations results also showed that a panel of 39 *loci* of similar variability to those used in the study would be needed to discriminate half-siblings from full-siblings or unrelated dyads with > 0.90 confidence probability.

**Table 1:** Summary of diversity statistics and power of set of markers used for relatedness analyses in cattle egret. Number of alleles (*Na*), observed heterozygosity (*Ho*), unbiased expected heterozygosity (*uHe*), Fixation Index (*F*_*IS*_), probability values of tests for deviation from Hardy-Weinberg Equilibrium (*PHWE*), probability of excluding a parent-pair (*P*_*E*_), probability of identity between siblings (*P*_*ID-Sibs*_) [54]. Multilocus estimates (± standard errors) are given in last row. Parameters were estimated using sample of unrelated females (*n* = 31) and males (*n* = 31).

*Locus*	*Na*	*Ho*	*uHe*	*F*_*IS*_	*PHWE*	*PE*	*P*_*ID-Sibs*_
***Bi01***	4	0.55	0.55	0.00	0.09	3.8E-01	3.0E-01
***Bi18***	4	0.25	0.25	0.00	0.99	2.2E-01	5.8E-01
***Bi20***	3	0.50	0.54	0.06	0.92	3.5E-01	3.3E-01
***Bi30***	3	0.22	0.20	-0.11	0.81	1.7E-01	6.6E-01
***Ah536***	7	0.64	0.66	0.02	1.00	6.0E-01	1.7E-01
***Bi32***	6	0.81	0.78	-0.04	0.28	7.5E-01	8.3E-02
***Bi33***	10	0.75	0.78	0.03	0.37	7.6E-01	8.3E-02
***Bi34***	7	0.69	0.64	-0.08	0.64	6.1E-01	1.7E-01
***Bi36***	7	0.80	0.75	-0.08	0.88	7.0E-01	1.1E-01
***Bi38***	4	0.72	0.64	-0.14	0.15	5.7E-01	1.8E-01
***Bi43***	5	0.70	0.59	-0.20	0.36	5.0E-01	2.3E-01
***Multilocus***	5.45 ± 0.65	0.60 ± 0.06	0.57 **±** 0.05		-	0.99	0.00064

### Parentage allocation

**Fig 2:**
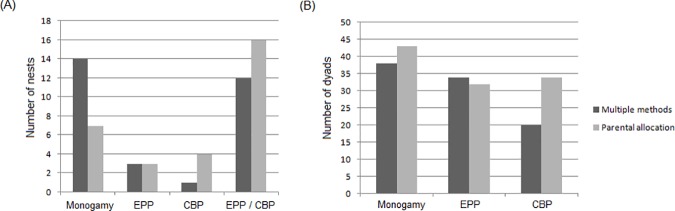
Proportion of inferred genetic mating system in cattle egret nests and nestling-dyads. (A) Number of nests showing genetic evidence of monogamy, extra-pair paternity (EPP), conspecific brood parasitism (CBP) or of both behaviors, as inferred by application of the parentage allocation procedure or the multiple methods approach; (B) Number of nestling-dyads inferred to be product of monogamy, EPP or CBP by both methods.

Three hundred one dyads from 31 nests were analyzed, among which 10.30% were adult dyads (i.e., mating partners), 59.14% were adult-nestling dyads (89 female nestlings and 89 male nestlings) and 30.56% were pairs of nestlings (*n* = 92). The conventional DNA-based parentage allocation method was initially applied to determine parentage by examining parent-offspring dyads in each nest. At the highest confidence level, Cervus simulations showed that attending males with a LOD score ≤ -2 and attending females with a LOD score of ≤ -1.9 were not directly related to the offspring being tested and could therefore be excluded as true fathers or true mothers, respectively. Using this threshold, the results of the parentage allocation analyses showed that 12 of the 89 nestlings (35.9%) were not related to their attending males and 34 (38.2%) were not related to their attending females (Table C in S1 File). Parentage allocation confirmed genetic monogamy in 48.3% of the adult-nestling pairs analyzed (Fig 2, Table C in S1 File). Among the 31 nests studied, the conventional DNA-based parentage allocation procedure revealed genetic monogamy in eight nests, EPP in three, CBP in four and both EPP and CBP in 16 nests (Table 2).

**Table 2:** Inferred genetic mating system of the cattle egret. Inferences drew using the parental allocation approach with samples from incubating adults or the multiple methods’ approach in absence of parental information. Inferred genetic mating system (i. e., monogamy, CBP: conspecific brood parasitism, or EPP: extra-pair paternity) refers to whole nests rather than dyads. Symbol ‘/’ indicates that both behaviors were detected in nest. ‘Not inferred’ (nest #16) indicates that the multiple methods approach could not reach a final diagnosis for the full brood. Detailed results of parentage and kinship analyses for all nests are given in Tables C and D in S1 File, respectively.

	Inferred genetic mating system	
Nests #	Parental allocation	Multiple methods
**1, 4, 6, 7, 27, 28, 31**	Monogamy	Monogamy
**2, 8, 9, 10, 11, 13, 14, 17, 18, 19. 25**	CBP / EPP	CBP / EPP
**30**	EPP	EPP
**3**	EPP	Monogamy
**5, 24**	CBP	Monogamy
**12**	CBP / EPP	CBP
**15, 21**	CBP	EPP
**16**	EPP	Not inferred
**20, 22, 23, 26**	CBP / EPP	Monogamy
**29**	Monogamy	CBP / EPP

### Relatedness and kinship classification

Using the multiple methods approach [11], consistent kinship patterns allowed us to infer the genetic mating system in 96.8% of the nests studied. Kinship patterns were resolved for 90 nestling dyads, 41.1% of which were full siblings, 37.8% were half siblings and 21.1% were unrelated. Inconsistent kinship patterns between dyads precluded a definitive determination of the genetic mating system in only one nest (#16, Table D in S1 File). In the group of 30 fully resolved nests, the approach of kinship classification lacking parental information inferred genetic monogamy in 14 nests, CBP in one nest, EPP in three nests and both behaviors in 12 nests (Table 2).

### Genetic mating system inferred in the presence or absence of parental information

Both the conventional parentage allocation method and the multiple methods approach (Fig A in S1 File) led to the same inference regarding the genetic mating system of the cattle egret in 19 out of 30 fully resolved nests (63.3%) (Table 2). Discrepancies between methods were found in eleven fully resolved nests (36.7%) and nine out of the eleven discordant cases were related to the inference of conspecific brood parasitism (nests 5, 24, 15, 20, 21, 22, 23, 26 and 29, Table 2). When genotypes of attending adults and offspring matched at most *loci*, both methods amply agreed in evidencing monogamy. When there were non-matching genotypes, the conventional likelihood-based parentage allocation method was more stringent, excluding more females or males and consequently inferring EPP or CBP for cases in which the multiple methods approach inferred monogamy (Table 2). The parental allocation method inferred a combined total rate of 74.2% of broods with alternative reproductive strategies. In contrast, the multiple methods approach of kinship classification lacking parental information inferred a combined total rate of 53.4% of broods with alternative reproductive strategies (Table 3). Considering only the 19 nests for which both approaches agreed, genetic monogamy was inferred in 38.8% and alternative reproductive strategies were detected in 61.2% (Table 2).

**Table 3:** Rates of alternative reproductive tactics inferred in wild waterbirds. Percentages of nests with evidence of extra-pair paternity (EPP) or conspecific brood parasitism (CBP) inferred in waterbirds studied to date with molecular tools. Inferences were made either by applying the parental allocation approach [57] or the multiple methods approach in the absence of parental information [11].

Species	Parental allocation		Multiple methods		Source
	EPP %	CBP %	EPP %	CBP %	
Roseate spoonbill	-	-	5.0%	24.0%	11
*Platalea ajaja*					
Wood stork	-	-	-	70.0%	11
*Mycteria americana*					
White-faced ibis	-	-	1.2%	13.7%	13
*Plegadis chihi*					
Jabiru stork	7.7%	-	7.1%	-	14
*Jabiru mycteria*					
Great cormorant	-	-	30.0%	-	16
*Phalacrocorax carbo sinensis*					
White stork	-	-	13.1%	-	17
*Ciconia ciconia*					
Cattle egret	61.3%	64.5%	50%	43.3%	This study
*Bubulcus ibis*					

## Discussion

### The ‘bug-egg’ method applied for parental sampling in a colonial tree-nesting bird

In the present study, we present a field protocol for the use of hematophagous insects contained in fake eggs to obtain blood samples from incubating adults in a cattle egret colony. This study demonstrates that it is feasible to obtain blood from incubating adults of colonial breeders, in exposed nests built next to each other in trees, offering a new path for researchers studying Ciconiiformes, or other understudied birds, in which parentage analyses were precluded (Table 3). Since its inception a decade ago for the study of ground-nesting seabirds [22], the ‘bug-egg’ method has been mostly applied to collect samples from the common tern [22–24, 64–68] and common swift [25], which are small birds with a markedly different biology from colonial egrets. More recently, this method was applied to sample adults of medium-sized raptors, such as the ground-nesting Montagu’s harrier [26] and the cavity-nesting Eurasian kestrel [27]. Those studies used the Mexican insect species *Dipetalogaster maximus*. The present study in the cattle egret represents the first use of *P*. *megistus*, which preferably feeds on birds [69], can be found in Brazil, Argentina, Uruguay, Paraguay and Bolivia [70], and it is therefore naturally adapted to the field conditions in the Neotropical region.

Blood obtained using *P*. *megistus* is a reliable source of DNA of sufficient quantity and useful for molecular sexing and microsatellites’ genotyping of cattle egrets. Blood ingested by Triatominae insects remains undigested after intake [21] making this collection method of special appeal for genetic studies, as DNA integrity maximizes the odds of success in amplifying molecular markers. Given that the negative controls in the present study yielded no amplification, we can be confident that contamination with Triatominae DNA is not a concern when using this method. Contamination between male and female blood was checked using a molecular sexing protocol. The fact that the ‘bug-eggs’ were left inside the nests for <20 min increased the odds of sampling only one adult per trial. The protocol used here enabled obtaining blood from incubating cattle egrets of both sexes at all the studied nest, similarly to previous studies in the common tern, which also exhibits biparental incubation [22–24, 64–68].

The results of the present study show that *P megistus* can be used as an alternative for sampling blood from elusive birds using the ‘bug-egg’ method. The sampling success rate (67.4%) was higher than rates achieved in some previous studies conducted with the common tern, the species for which the method was developed (34.0% in [22], 40.0% in [24]), but lower than rates reported in other studies (80.0% in [64], 86.1% in [67, 68]). Sampling success rates using the ‘bug-egg’ method are influenced by many factors and therefore vary across species, studies and climates. For example, the starvation period to which the insects are subjected prior to field sampling intensifies their willingness to feed [21] (see Table 1 in [27]) thereby affecting both the trial duration and sampling success rate. In the present study, 15 days of starvation were enough for *P*. *megistus* larvae to suck blood from cattle egrets in a few minutes in most trials. Trial duration is another influential factor to the success of the ‘bug-egg’ method. We reached sampling success of 67.4% with 20 min trials for collecting blood from cattle egrets nesting in trees. The trial duration in previous studies using *D*. *maximus* ranged from 30–60 min [25, 65] to 12 h [27]. Differences in trial duration between studies could be inherent to the biology of the different species of Triatominae used, but may also be explained by the influence of climatic factors, such as temperature and humidity, on the feeding behavior of insects. The present study was conducted in a subtropical environment, whereas previous studies used *D*. *maximus* to sample blood from terns, swifts, harriers and kestrels were conducted in colder climates [22–27, 64–68]. At warmer conditions, insects are more active and may be more willing to feed. Hence, the shorter trial duration and shorter starvation times needed for *P*. *megistus* to feed on the blood of cattle egrets could be a consequence of warmer, more humid conditions of the sampling site. Further studies are needed for an in-depth comparison of the performances of both insect species under equal field conditions.

### Genetic mating system of the cattle egret: inferences made in the presence or absence of parental information

When candidate parents are available, inferences on the genetic mating system of bird species have traditionally been made using DNA-based approaches aimed at either attributing parentage to the individuals observed incubating the eggs and caring for offspring or excluding these individuals as parents [5]. Such analyses are based on the simple, strong concept that, for a codominant marker under Mendelian inheritance, parents and offspring must share at least one allele at every *locus* [5, 56]. When parental genotypes are not available, however, the reproductive behavior of adults has been mostly inferred by observing the relatedness arrays in the progeny [4, 5, 11]. It is noteworthy that this approach has revealed the occurrence of alternative reproductive tactics in most species of waterbirds (Ciconiiformes, Suliformes) studied so far using this method of kinship classification of nestlings. Given that elusive reproductive adults in this group of birds are difficult to sample at nests, the application of the powerful method of parental allocation has been limited to the occasional sampling of feathers either in nests or on the ground below [14]. The parental allocation method has also been applied to address the genetic mating system of some waterbird species in captivity, using parental blood samples that were available as part of routine animal health examination procedures [11, 15]. Conclusions reached in captivity, however, cannot be extrapolated to natural populations.

We overcame the parental sampling limitation by using the ‘bug-egg’ method (as described in the previous section), and obtained valuable samples from incubating adults in a wild breeding colony of cattle egrets. We were able, then, to perform conventional parentage allocation analyses to either confirm or exclude attending females or attending males, and reach to reliable estimates of CBP and EPP rates in the nests studied [58]. In parallel, interested in comparing the inferences on the genetic mating systems made in the presence and absence of parental information using the same set of familial genotypes, we applied the conservative multiple methods approach of kinship classification of nestlings in nests [11]. The results revealed that both analytical approaches generally agreed in evidencing alternative reproductive tactics, but the rates of inferred alternative behaviors varied depending on the method used (Table 2, Fig 2). Overall, the conventional likelihood-based parentage allocation method inferred almost half the rate of genetic monogamy than the multiple methods approach of kinship classification. As expected, the multiple methods approach lacking parental information was more conservative, inferring monogamy in nests in which the parental allocation method inferred alternative strategies (Fig 2, Tables 2 and 3). Using the multiple methods approach alone, the results of the simulations (Fig A in S1 File) demonstrated sufficient power to distinguish genetic monogamy, which produces full-siblings, from conspecific brood parasitism, which is inferred when unrelated nestlings are found inside a nest. Alternative reproductive tactics such as EPP and CBP were also detected with both methods, although at different rates (Tables 2 and 3). We found that the more powerful approach of parental allocation inferred 1.39 times higher rates of EPP and CBP than the approach lacking parental information (Table 3). These results indicate that the rates of alternative reproductive strategies inferred in the absence of parental information could be underestimated. Moreover, given the limited power of the multiple methods approach to discriminate half-siblings from unrelated nestlings or full-siblings (Fig A in S1 File), caution should be exercised regarding the inference of EPP based on this method alone. In the present study, this situation occurred with three nests (#15, #21, and #30) in which the kinship classification method inferred EPP by the presence of half-siblings, whereas the parentage allocation method inferred either CBP (#15 and #21) or EPP (#30). Taken together, the present results indicate that relying only on the multiple methods approach of kinship classification could lead to biased inferences on the genetic mating system of the species of interest. This method could still be applied as an exploratory analysis in the absence of parental information or ancillary behavioral data, but its limitations, as revealed in the present study, should be taken into account when drawing conclusions. Moreover, simulations showed that reaching > 90 confidence in discriminating adjacent relationships with the kinship method alone requires isolating additional 28 *loci* of similar variability to the ones used here. When working with a wild non-model organism such as the cattle egret, isolating new markers would be more difficult, expensive and time-consuming than sampling elusive candidate parents using the ‘bug-egg’ method. Hence, this method seems to be a very appealing strategy when the goal is to increase confidence in genetic mating system inference.

The combined use of a minimally invasive sampling method and genetic parentage allocation tests allowed us to more adequately characterize the genetic mating system of the invasive cattle egret. In line with previous behavioral evidence reporting extra-pair copulation and conspecific brood parasitism in this species [8, 42, 43, 71–74], we found strong support for a non-monogamous genetic mating system. The more powerful parental allocation approach identified a combined rate of 74.2% of extra-pair paternity and conspecific brood parasitism (Table 3). The scarcity of studies using this method with waterbirds (Table 3) prevents further comparisons between rates of alternative reproductive tactics among species. Regardless of the method employed, however, the rates of EPP and CBP identified in the present study are among the highest reported for waterbirds (Table 3). One may therefore speculate that the ‘bug-egg’ method applied to sample candidate parents in other species of this avian group could reveal higher rates of alternative reproductive tactics.

Extra-pair paternity and conspecific brood parasitism can have variable consequences for the stability and survival of populations, depending on ecological conditions and life history traits [6, 7]. For example, in populations of invasive species outside their native ranges, such as the one studied herein, extra-pair paternity can counteract the negative effects of inbreeding [75]. EPP could also increase the breeding success of male cattle egrets, which would promote the choice of females for promiscuous mates as a way of increasing the fitness of the offspring [38]. Moreover, CBP in colonial birds can increase average population fitness and become a stable evolutionary strategy when resources for nesting and foraging are limited [7]. In species with siblicidal infanticide, such as the cattle egret [76], CBP may have evolved as an adaptive strategy used by parents to maximize offspring survival by eliminating competition between related nest mates [77]. Further studies are needed to investigate whether this hypothesis applies to the cattle egret as well.

The novel information on the genetic mating system of cattle egrets revealed in this study also has important implications for the management of populations of this increasingly expanding invasive bird. Managers can now reliably equate the observed reproductive behavior of adults to the underlining genetic mating system of the species to plan more effective control techniques. Research in other breeding populations of the cattle egret in both its native and non-native range is needed to gain a better understanding of the correlates among the flexible genetic mating system, population survival and invasive potential.

## Conclusions

This study presents the first application of the ‘bug-egg’ method to inspect the genetic mating system of a colonial tree-nesting bird. We used a field protocol and validated the use of the *P*. *megistus* (Triatominae) for collecting blood from incubating cattle egrets. We recommend that researchers planning to use this method on other birds carry out pilot sampling to evaluate the acceptance of fake eggs by incubating adults as well as determine both the number and duration of trials needed to sample both sexes and achieve acceptable sampling success rates. The baseline methodological guidelines provided here can be applied to collect blood samples from elusive adults of other colonial waterbirds.

We found evidence supporting our hypotheses of the occurrence of alternative reproductive tactics concurrently with genetic monogamy in the cattle egret, which is in line with data from behavioral observations. By performing comparative analyses based on the same familial dataset, a major finding from this study was that inferences on the genetic mating system made in the absence of parental information could underestimate the rates of alternative reproductive tactics and should therefore be interpreted with caution. Thus, we encourage researchers to invest more in obtaining samples from potential parents to enable an adequate assessment of the mating system of the species of interest.

In sum, this study contributes to the growing body of evidence supporting the occurrence of non-monogamous mating systems in non-passerine birds. Using a more powerful methodological framework, we showed that wild cattle egrets have a flexible, complex genetic mating system that includes monogamy as well as extra-pair paternity and conspecific brood parasitism. Finally, we expand knowledge on the genetic mating system of colonial waterbirds, enabling a better understanding of the evolutionary ecology of variable mating tactics in this group of organisms, which have been poorly studied.

## Supporting information

S1 File Appendix A, Fig A, Tables A-D.
(PDF)
