# Supplementary material for: Use of noninvasive ‘bug-eggs’ to enable comparative inferences on genetic mating system with and without parental information: A study in a cattle egret colony
Source: PLoS One. 2017 Aug 30;12(8):e0183153. doi: 10.1371/journal.pone.0183153 (PMC5576647; doi:10.1371/journal.pone.0183153)
Supplement: S1 File — Appendix A, Fig A, Tables A-D. (PDF) [file pone.0183153.s001.pdf]

Supporting Information

Use of noninvasive 'bug-eggs' to enable comparative inferences on genetic mating system with and without parental information: a study in a cattle egret colony

Carolina Isabel Miño[1,¶,*], Elaine Dantas de Souza[2,&], Emmanuel Moralez-Silva[2,&] Talita Alvarenga Valdes[2,&], Vera Lúcia Cortiço Corrêa Rodrigues[3,&], and Sílvia Nassif Del Lama[2,¶]

[1] Instituto de Biología Subtropical (IBS), Nodo Iguazú, Universidad Nacional de Misiones (UNaM) – CONICET, Puerto Iguazú, Misiones, Argentina.

[2] Departamento de Genética e Evolução, Universidade Federal de São Carlos, São Carlos-SP, Brazil.

[3] Superintendência de Controle de Endemias, Mogi-Guaçu, SP, Brazil.

* Corresponding author; E-mail: carolinamino@conicet.gov.ar (CIM)

Appendix A. Amplification conditions for microsatellite markers.

Polymerase Chain Reaction (PCR) amplifications were carried out in a final volume of 10 µl containing 1x buffer [100 mM Trizma®-HCl (pH 8.3, 25°C), 500 mM KCl], 25 mM $MgCl_2$, 0.5 U of *Taq* DNA polymerase (Sigma-Aldrich), 0.25 mM of dNTPs (Amersham Biosciences) and 20 ng of genomic DNA as template. Dynamic labelling of primers was used with the addition of an M13-tail at the end of *forward* primers, so that PCR mixes included 2 pmol of species-specific M13-tailed *forward* primers (Alpha DNA, Montreal, Canada), 8 pmol of specific *reverse* primer and 8 pmol of M13 primer with a fluorescent tag (either HEX, 6-FAM, or TET). PCRs were performed under the following cycling parameters: an initial denaturation at 94 °C for 5 min following with 30 cycles of 94 °C for 30 s, ramp 1 °C/s to primer annealing temperature (see Table S1 below), 45 s at that temperature, ramp 1 °C/s to 72 °C, 72 °C for 45 s, followed by 10 cycles of denaturation at 94 °C for 30 s, ramp 1 °C/s to 53°C, 53°C for 45 s (M13 primer annealing), ramp 1 °C/s to 72 °C, extension at 72 °C for 45 s, and a final extension at 72 °C for 10 min. PCRs were carried out in an Veriti® thermal cycler (Applied Biosystems®).

Fig A. Power of the set of microsatellites for relationship inference in cattle egrets. Power Index for Relationship Inference as function of the number of *loci* used, resulting from simulation conducted in KinInfor v.2 software [56] adding *loci*, one at a time, starting from the better ranked *locus* up to all 11 *loci*. PO: parent-offspring, FS: full-siblings, HS: half-siblings, UR: unrelated dyads.

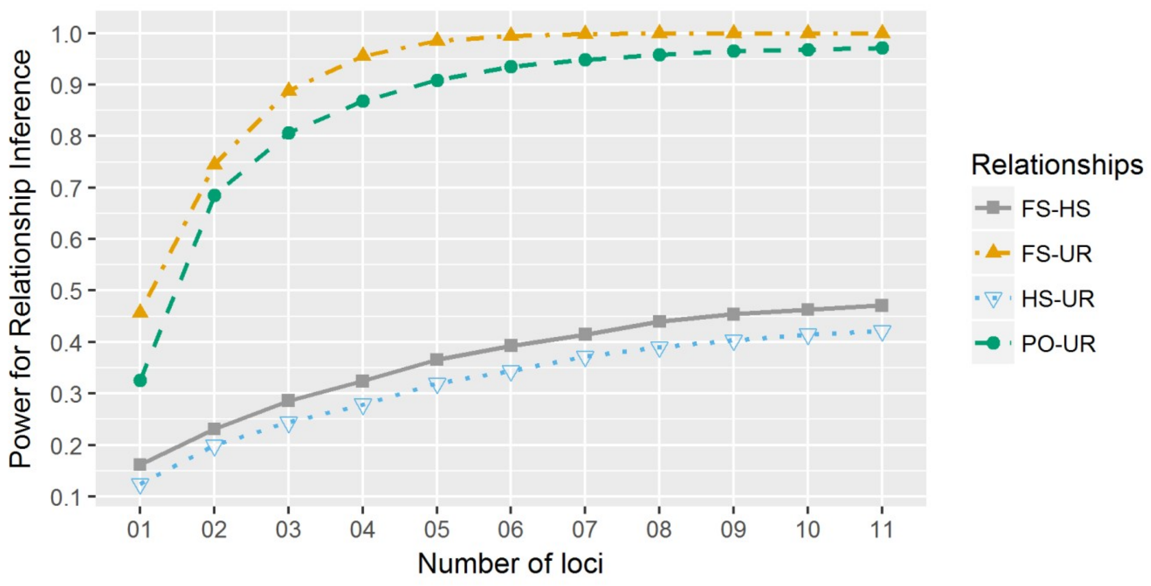

Table A. Description of primers and fragments amplified in cattle egret.

Names, *forward* primer sequences, repeat motifs, annealing temperature (*Ta*) and size ranges of amplified fragments for 14 microsatellite *loci* analyzed, including six new species-specific *loci*.

Locus	*Sequence 5'- 3'*	*Repeat motif*	*Ta (˚C)*	*Size range (pb)*	*Source*
Bi01	GAGCCCAGTGAATTGTTTAG	$(GT)_{11}$	57	245-251	[49]
Bi15	GGGCTTGTATGATGAACTTT	$(TTCC)_2(AG)_4$	56	182-184	[49]
Bi18	CATGACCATGTTCCTTCG	$(TA)_8(CA)_6$	57	190-196	[49]
Bi20	TGGATTAGGTCCTGTTATTC	$(TGC)_5$	56	232-238	[49]
Bi26	GTCTCAGACCACCCGTG	$(AG)_9$	59	269-273	[49]
Bi28	TCTTAACAGATGTTCCAAGTG	(TG)8	56	192-194	[49]
Bi30	ACCTTAGCAAACCCCCTC	$(GT)_7$	60	204-210	[49]
Ah536	CCTGGTTTAGAAGATCACATGATGGAG	$(AC)_{14}$	58	94-110	[40]
Bi32	GCTAAATTGCAGCTGTGGGA	(AG)	59	164-198	This study
Bi33	GGCAATGAAGACTGAAATGTGC	(AC)	57	175-203	This study
Bi34	AAACTCTGCAACTCGCACAAA	(AC)	55	93-111	This study
Bi36	GTGTGGCCGCTCTTCTTTC	(AC)	56	82-102	This study
Bi38	TGCTGTTTAAATGCTGGTGC	(AG)	55	182-190	This study
Bi43	AAATTGGTCAGCAGTGGGTG	(AC)	54	122-130	This study

Table B. Summary statistics of microsatellite diversity and informative power of full set of markers tested in cattle egret.
Parameters estimated using 62 unrelated adults of both sexes. *Na*: No. of alleles; *Ho*: Observed Heterozygosity; *uHe*: Unbiased Expected Heterozygosity; F_{IS}: Fixation Index; *PHWE*: Probability of tests for deviation from Hardy-Weinberg Equilibrium; P_E: probability of exclusion when only one parent is known; $P_{ID\text{-}Sib}$: multilocus probability of identity between siblings [54, 55]. Asterisks denote significance after Bonferroni correction ($P < 0.001$). Multilocus estimates (± standard errors) given in last row.

Locus	*Na*	*Ho*	*uHe*	F_{IS}	*PHWE*	*PE*	$P_{ID\text{-}Sibs}$
Bi01	4	0.55	0.55	0	0.09	0.33	0.55
Bi15	2	0.50	0.49	-0.03	0.84	0.11	0.60
Bi18	4	0.25	0.25	0	0.99	0.33	0.77
Bi20	3	0.50	0.54	0.06	0.92	0.22	0.57
Bi26	3	0.14	0.16	0.12	0.00***	0.22	0.85
Bi28	2	0.14	0.13	-0.08	0.55	0.11	0.87
Bi30	3	0.22	0.20	-0.11	0.81	0.22	0.82
Ah536	7	0.64	0.66	0.02	1	0.54	0.46
Bi32	6	0.81	0.78	-0.04	0.28	0.49	0.38
Bi33	10	0.75	0.78	0.03	0.37	0.66	0.38
Bi34	7	0.69	0.64	-0.08	0.64	0.54	0.47
Bi36	7	0.80	0.75	-0.08	0.88	0.54	0.41
Bi38	4	0.72	0.64	-0.14	0.15	0.33	0.48
Bi43	5	0.70	0.59	-0,2	0.36	0.42	0.52
Multilocus	4.79 ± 0.62	0.53 ± 0.07	0.51 ± 0.06		-	0.99	0.0002

Table C. Results of parentage allocation tests carried out in each of 31 cattle egret nests from a colony located in Rio Claro, SP, Brazil. Attending female and male are shown for each nest together with the likelihood scores (LOD, scientific notation) obtained for each adult-offspring pair. Attending adults with LOD scores ≥ to threshold values (-1.9 for females and -2 for males) were confirmed as dams or sires of the offspring being tested. Last column indicates the inferred mating system of adults, given the parental allocation results for all offspring within each nest. Inferences of extra-pair paternity (EPP) and conspecific brood parasitism (CBP) are shaded in grey.

Nest #	Offspring	Attending female	Maternity	LOD	Attending male	Paternity	LOD	Mating system
**1**	F3.1	P3D	Confirmed	2.67E+14	P3C	Confirmed	5.66E+14	Monogamy
	F3.2	P3D	Confirmed	5.87E+14	P3C	Confirmed	4.25E+14	Monogamy
**2**	F5.1	P5C	Confirmed	4.90E+14	P5D	Confirmed	7.26E+14	Monogamy
	F5.2	P5C	Confirmed	5.00E+14	P5D	Confirmed	1.87E+14	Monogamy
	F5.3	P5C	Not-Assigned	-5.88E+14	P5D	Not-Assigned	-5.37E+13	EPP/CBP
**3**	F12.1	P12B	Confirmed	1.08E+14	P12A	Not-Assigned	-6.36E+14	EPP
	F12.2	P12B	Confirmed	-9.09E+14	P12A	Not-Assigned	-4.83E+14	EPP
**4**	F14.1	P14C	Confirmed	3.98E+14	P14D	Confirmed	7.31E+14	Monogamy
	F14.2	P14C	Confirmed	4.19E+14	P14D	Confirmed	6.23E+14	Monogamy
**5**	F22.1	P22E	Not-Assigned	-9.22E+12	P22D	Confirmed	1.61E+14	CBP
	F22.2	P22E	Not-Assigned	-7.50E+12	P22D	Confirmed	2.22E+14	CBP
**6**	F58.1	P58A	Confirmed	3.16E+14	P58B	Confirmed	4.34E+14	Monogamy
	F58.2	P58A	Confirmed	5.34E+14	P58B	Confirmed	1.94E+14	Monogamy
**7**	F85.1	P85D	Confirmed	7.29E+14	P85C	Confirmed	6.31E+14	Monogamy
	F85.2	P85D	Confirmed	2.63E+14	P85C	Confirmed	5.06E+14	Monogamy
**8**	F93.1	P93A	Confirmed	2.76E+14	P93B	Confirmed	5.02E+14	Monogamy
	F93.2	P93A	Confirmed	6.13E+14	P93B	Confirmed	1.81E+14	Monogamy
	F93.3	P93A	Not-Assigned	-5.88E+14	P93B	Not-Assigned	-1.71E+15	EPP/CBP
**9**	F132.1	P132B	Confirmed	5.55E+14	P132A	Not-Assigned	-3.41E+13	EPP
	F132.2	P132B	Confirmed	2.87E+14	P132A	Confirmed	4.55E+14	Monogamy
	F132.3	P132B	Confirmed	6.29E+14	P132A	Confirmed	2.09E+14	Monogamy
	F132.4	P132B	Not-Assigned	-8.98E+14	P132A	Confirmed	4.96E+14	CBP
**10**	F152.1	P152A	Confirmed	2.68E+14	P152B	Confirmed	5.76E+14	Monogamy
	F152.2	P152A	Not-Assigned	-8.37E+14	P152B	Not-Assigned	-3.63E+14	EPP/CBP
	F152.3	P152A	Confirmed	2.99E+14	P152B	Confirmed	6.49E+14	Monogamy
	F152.4	P152A	Confirmed	1.87E+14	P152B	Not-Assigned	-9.49E+14	EPP

**11**	F191.1	P191A	Not-Assigned	5.91E+13	P191B	Not-Assigned	-5.63E+14	EPP/CBP
	F191.2	P191A	Confirmed	4.84E+14	P191B	Confirmed	3.18E+14	Monogamy
	F191.3	P191A	Not-Assigned	-3.22E+14	P191B	Not-Assigned	-4.52E+14	EPP/CBP
	F191.4	P191A	Not-Assigned	-3.89E+14	P191B	Not-Assigned	-5.19E+14	EPP/CBP
**12**	F198.1	P198A	Not-Assigned	-6.63E+14	P198C	Confirmed	1.07E+15	CBP
	F198.3	P198A	Not-Assigned	-4.59E+14	P198C	Not-Assigned	-4.06E+14	EPP/CBP
**13**	F199.1	P199.a	Not-Assigned	-1.15E+15	P199.b	Not-Assigned	-3.13E+14	EPP/CBP
	F199.3	P199.a	Confirmed	5.99E+14	P199.b	Confirmed	1.99E+14	Monogamy
	F199.4	P199.a	Not-Assigned	-5.69E+14	P199.b	Not-Assigned	-2.74E+14	EPP/CBP
**14**	F204.1	P204C	Not-Assigned	1.05E+14	P204A	Confirmed	4.26E+14	CBP
	F204.2	P204C	Not-Assigned	-3.50E+14	P204A	Not-Assigned	-1.25E+15	EPP/CBP
	F204.3	P204C	Not-Assigned	-4.25E+14	P204A	Not-Assigned	-1.28E+15	EPP/CBP
**15**	F211.1	P211A	Not-Assigned	-2.76E+14	P211D	Confirmed	2.19E+14	CBP
	F211.2	P211A	Confirmed	5.33E+14	P211D	Confirmed	2.50E+14	Monogamy
	F211.3	P211A	Not-Assigned	1.28E+14	P211D	Confirmed	3.78E+14	CBP
	F211.4	P211A	Not-Assigned	-3.14E+14	P211D	Confirmed	5.36E+14	CBP
**16**	F212.1	P212A	Confirmed	3.00E+14	P212B	Not-Assigned	1.53E+13	EPP
	F212.2	P212A	Confirmed	3.86E+14	P212B	Not-Assigned	1.52E+14	EPP
	F212.3	P212A	Confirmed	4.85E+14	P212B	Confirmed	4.02E+14	Monogamy
**17**	F217.1	P217C	Confirmed	3.14E+14	P217A	Confirmed	3.17E+14	Monogamy
	F217.2	P217C	Not-Assigned	-3.60E+13	P217A	Not-Assigned	-2.15E+14	EPP/CBP
	F217.3	P217C	Not-Assigned	-9.73E+13	P217A	Not-Assigned	1.46E+13	EPP/CBP
	F217.4	P217C	Not-Assigned	1.13E+13	P217A	Not-Assigned	-8.47E+14	EPP/CBP
**18**	F220.2	P220A	Confirmed	6.93E+14	P220C	Confirmed	2.59E+14	Monogamy
	F220.3	P220A	Not-Assigned	-5.37E+14	P220C	Not-Assigned	-8.06E+14	EPP/CBP
	F220.4	P220A	Not-Assigned	-9.27E+13	P220C	Not-Assigned	-6.88E+14	EPP/CBP
**19**	F226.1	P226A	Confirmed	6.95E+14	P226C	Confirmed	1.83E+14	Monogamy
	F226.2	P226A	Not-Assigned	1.45E+14	P226C	Not-Assigned	-7.72E+14	EPP/CBP
	F226.3	P226A	Confirmed	6.73E+14	P226C	Not-Assigned	-3.19E+13	EPP
**20**	F234.1	P234A	Confirmed	4.51E+14	P234D	Confirmed	1.75E+14	Monogamy
	F234.2	P234A	Confirmed	5.74E+14	P234D	Not-Assigned	-3.78E+14	EPP
	F234.3	P234A	Not-Assigned	1.51E+13	P234D	Confirmed	2.52E+14	CBP
**21**	F239.1	P239D	Confirmed	5.65E+14	P239A	Confirmed	5.17E+14	Monogamy

	F239.2	P239D	Confirmed	3.78E+14	P239A	Confirmed	3.70E+14	Monogamy
	F239.3	P239D	Not-Assigned	4.15E+13	P239A	Confirmed	2.95E+14	CBP
**22**	F241.1	P241.b	Confirmed	3.38E+14	P241.a	Confirmed	6.37E+14	Monogamy
	F241.2	P241.b	Confirmed	4.62E+14	P241.a	Confirmed	2.17E+14	Monogamy
	F241.3	P241.b	Not-Assigned	-1.52E+14	P241.a	Not-Assigned	-8.68E+13	EPP/CBP
	F241.4	P241.b	Confirmed	2.80E+14	P241.a	Confirmed	3.05E+14	Monogamy
**23**	F244.1	P244A	Not-Assigned	1.03E+14	P244C	Confirmed	7.47E+14	CBP
	F244.2	P244A	Confirmed	4.14E+14	P244C	Not-Assigned	1.29E+14	EPP
	F244.3	P244A	Confirmed	5.36E+14	P244C	Confirmed	5.14E+14	Monogamy
**24**	F250.1	P250A	Not-Assigned	1.46E+14	P250B	Confirmed	4.18E+14	CBP
	F250.2	P250A	Not-Assigned	-4.02E+13	P250B	Confirmed	5.60E+14	CBP
**25**	F252.1	P252D	Confirmed	4.72E+14	P252A	Not-Assigned	-6.64E+12	EPP
	F252.2	P252D	Confirmed	7.86E+14	P252A	Confirmed	4.92E+14	Monogamy
	F252.3	P252D	Not-Assigned	1.36E+14	P252A	Confirmed	8.16E+14	CBP
**26**	F253.1	P253.c	Confirmed	7.09E+14	P253.a	Confirmed	6.99E+14	Monogamy
	F253.2	P253.c	Not-Assigned	-1.54E+15	P253.a	Not-Assigned	-7.70E+14	EPP/CBP
	F253.3	P253.c	Confirmed	7.47E+14	P253.a	Confirmed	7.58E+14	Monogamy
	F253.5	P253.c	Not-Assigned	-6.15E+14	P253.a	Not-Assigned	-5.05E+14	EPP/CBP
**27**	F254.1	P254.b	Confirmed	8.05E+14	P254.a	Confirmed	7.55E+14	Monogamy
	F254.2	P254.b	Confirmed	5.34E+14	P254.a	Confirmed	9.49E+14	Monogamy
	F254.3	P254.b	Confirmed	6.78E+14	P254.a	Confirmed	7.15E+14	Monogamy
**28**	F256.1	P256.a	Confirmed	8.09E+14	P256.b	Confirmed	4.58E+14	Monogamy
	F256.2	P256.a	Confirmed	2.73E+14	P256.b	Confirmed	2.31E+14	Monogamy
	F256.3	P256.a	Confirmed	5.60E+14	P256.b	Confirmed	6.35E+14	Monogamy
**29**	F76.1	P76B	Confirmed	3.36E+14	P76D	Confirmed	4.47E+14	Monogamy
	F76.2	P76B	Confirmed	3.36E+14	P76D	Confirmed	4.47E+14	Monogamy
**30**	F74.1	P74A	Confirmed	4.60E+14	P74B	Not-Assigned	-6.35E+13	EPP
	F74.2	P74A	Confirmed	3.88E+14	P74B	Not-Assigned	-7.94E+14	EPP
**31**	F209.1	P209C	Confirmed	3.53E+14	P209A	Confirmed	5.14E+14	Monogamy
	F209.2	P209C	Confirmed	2.52E+14	P209A	Confirmed	5.27E+14	Monogamy

Table D. Genetic mating system inferred in each of 31 cattle egret nests from a colony located in Rio Claro, SP, Brazil. Attending females in each nest are shown in italics, attending males in bold type and nestlings in regular font. Relatedness values (Q&G *R*), most-likely relationship hypothesis, probability of being assigned to same sib-group (COLONY *P*), probability of being assigned to same cluster (PEDIGREE *P*), and kinship classification shown for each pair.

Nest #	Pair	Q&G *R*	ML-Relate R	COLONY *P*	PEDIGREE *P*	Classified as...	Mating System
1	**P3C-** *P3D*	-0.157	UR	NC	0.002	Unrelated	
	**P3C-** F3.1	0.554	PO	1	18.529	Parent-offspring	
	P3D- F3.1	0.326	PO	1	4.56	Parent-offspring	Genetic monogamy
	**P3C-** F3.2	0.483	PO	1	8.06	Parent-offspring	
	P3D- F3.2	0.455	PO	1	190.58	Parent-offspring	
	F3.1- F3.2	0.531	FS	1	47.16	Full-siblings	
	P5C- **P5D**	-0.010	UR	NC	0.067	Unrelated	
2	*P5C*- F5.1	0.491	PO	1	28.87	Parent-offspring	
	**P5D-** F5.1	0.507	PO	1	18.1	Parent-offspring	
	P5C- F5.2	0.620	PO	1	175.3	Parent-offspring	
	**P5D-** F5.2	0.448	PO	1	3.9	Parent-offspring	EPP/CBP
	F5.1- F5.2	0.661	FS	1	207.16	Full-siblings	
	P5C- F5.3	0.241	UR	1	0.21	Unrelated	
	**P5D-** F5.3	0.167	HS	NA	1.19	Half-siblings	
	F5.1- F5.3	-0.001	UR	1	0.076	Half-siblings	
	F5.2- F5.3	0.383	FS	1	1.91	Half-siblings	
3	**P12A-** *P12B*	0.435	HS	NC	0.92	Unrelated	
	**P12A-** F12.1	0.845	FS	NA	3.14	Parent-offspring	
	P12B-F12.1	0.504	PO	0.98	450.38	Parent-offspring	
	**P12A-** F12.2	0.438	PO	NA	0.01	Parent-offspring	Genetic monogamy
	P12B- F12.2	-0.056	UR	0.39	0.12	Parent-offspring	
	F12.1- F12.2	0.568	FS	1	21.47	Full-siblings	
	P14C- **P14D**	-0.056	UR	NC	0.07	Unrelated	
	P14C- F14.1	0.383	PO	1	8.99	Parent-offspring	
4	**P14D-** F14.1	0.452	PO	1	61.94	Parent-offspring	Genetic monogamy
	P14C- F14.2	0.433	PO	1	16.33	Parent-offspring	

	**P14D-** F14.2	0.505	PO	1	119.1	Parent-offspring	
	F14.1- F14.2	0.369	FS	1	58.8	Full-siblings	
	**P22D-** *P22E*	-0.152	UR	NC	0.01	Unrelated	
**5**	**P22D-** F22.1	0.295	PO	0.6	0.48	Parent-offspring	
	P22E- F22.1	0.406	HS	1	2.26	Parent-offspring	Genetic monogamy
	**P22D-** F22.2	0.314	PO	0.6	1.76	Parent-offspring	
	P22E- F22.2	0.469	PO	1	14.67	Parent-offspring	
	F22.1- F22.2	0.780	FS	1	309.88	Full-siblings	
	P58A- **P58B**	0.251	UR	NC	0.15	Unrelated	
**6**	*P58A*- F58.1	0.704	PO	1	508.07	Parent-offspring	
	**P58B-** F58.1	0.609	PO	1	41.77	Parent-offspring	Genetic monogamy
	P58A- F58.2	0.735	PO	1	586.05	Parent-offspring	
	**P58B-** F58.2	0.419	PO	1	81.88	Parent-offspring	
	F58.1- F58.2	0.485	FS	1	49.82	Full-siblings	
	**P85C-** *P85D*	0.234	HS	NC	1.9	Unrelated	
**7**	**P85C-** F85.1	0.553	PO	1	40.96	Parent-offspring	
	P85D- F85.1	0.670	PO	1	1117.36	Parent-offspring	Genetic monogamy
	**P85C-** F85.2	0.784	PO	1	347.41	Parent-offspring	
	P85D- F85.2	0.532	PO	1	12.88	Parent-offspring	
	F85.1- F85.2	0.540	FS	1	30.93	Full-siblings	
**8**	*P93A*- **P93B**	0.038	HS	NC	0.03	Unrelated	
	P93A- F93.1	0.361	PO	1	3.61	NA	
	**P93B-** F93.1	0.492	PO	1	22.88	Parent-offspring	
	P93A- F93.2	0.588	PO	1	79.17	Parent-offspring	
	**P93B-** F93.2	0.297	PO	1	0.26	NA	
	F93.1- F93.2	0.387	HS	1	2.85	Half-siblings	
	P93A- F93.3	0.240	HS	NA	0.37	Unrelated	EPP/CBP
	**P93B-** F93.3	-0.225	UR	NA	0.00	Unrelated	
	F93.1- F93.3	0.021	UR	NA	0.3	Unrelated	
	F93.2- F93.3	0.107	UR	NA	0.69	Unrelated	
	**P132A-** *P132B*	-0.357	UR	NC	0	Unrelated	
	**P132A-** F132.1	0.258	HS	1	0.37	NA	
**9**	*P132B*- F132.1	0.164	PO	1	34.79	Parent-offspring	

	**P132A-** F132.2	0.332	PO	1	10.42	Parent-offspring	
	P132B- F132.2	0.266	PO	1	2.06	NA	
	F132.1- F132.2	0.238	FS	1	4.14	Half-siblings	
	**P132A-** F132.3	0.255	PO	1	1.18	NA	EPP/CBP
	P132B- F132.3	0.346	PO	1	229	Parent-offspring	
	F132.1- F132.3	0.328	FS	1	2096.53	Full-siblings	
	F132.2- F132.3	0.328	FS	1	75.45	Full-siblings	
	**P132A-** F132.4	0.319	PO	1	13.98	Parent-offspring	
	P132B- F132.4	-0.085	HS	NA	0.15	Unrelated	
	F132.1- F132.4	-0.212	HS	1 hs	1.02	Half-siblings	
	F132.2- F132.4	-0.078	HS	1 hs	10.13	Half-siblings	
	F132.3- F132.4	0.048	HS	1 hs	4.04	Half-siblings	
	P152A- **P152B**	0.140	UR	NC	0.13	Unrelated	
**10**	*P152A*- F152.1	0.500	PO	1	10.69	Parent-offspring	
	**P152B-** F152.1	0.423	PO	1	102.67	Parent-offspring	
	P152A- F152.2	-0.024	UR	NA	0.13	Unrelated	
	**P152B-** F152.2	0.112	UR	NA	0.32	Unrelated	
	F152.1- F152.2	-0.147	UR	NA	0.16	Unrelated	EPP/CBP
	P152A- F152.3	0.598	PO	1	80.41	Parent-offspring	
	**P152B-** F152.3	0.419	PO	1	125.18	Parent-offspring	
	F152.1- F152.3	0.590	FS	1	1173.88	Full-siblings	
	F152.2- F152.3	0.265	FS	NA	2.26	Half-siblings	
	P152A- F152.4	0.263	PO	NA	1.97	Unrelated	
	**P152B-** F152.4	-0.239	UR	0.047	0.008	Unrelated	
	F152.1- F152.4	0.015	UR	NA	0.16	Unrelated	
	F152.2- F152.4	-0.241	UR	NA	0.05	Unrelated	
	F152.3- F152.4	0.007	UR	NA	0.15	Unrelated	
	P191A- **P191B**	0.370	FS	NC	2.53	Full-siblings	
**11**	*P191A*- F191.1	0.478	HS	NA	7.62	Unrelated	
	**P191B-** F191.1	0.387	HS	NA	0.87	Unrelated	
	P191A- F191.2	0.607	PO	0.66	49.09	Parent-offspring	
	**P191B-** F191.2	0.583	PO	NA	26.55	Parent-offspring	
	F191.1- F191.2	0.515	FS	NA	22.14	Full-siblings	
	P191A- F191.3	0.042	UR	NA	0.06	Unrelated	EPP/CBP

	**P191B-** F191.3	0.121	UR	NA	0.031	Unrelated	
	F191.1- F191.3	0.065	UR	NA	0.029	Unrelated	
	F191.2- F191.3	0.092	UR	NA	0.06	Unrelated	
	P191A- F191.4	0.042	UR	NA	0.06	Unrelated	
	**P191B-** F191.4	0.121	UR	NA	0.031	Unrelated	
	F191.1- F191.4	0.065	UR	NA	0.029	Unrelated	
	F191.2- F191.4	0.092	UR	NA	0.06	Unrelated	
	F191.3- F191.4	1.000	FS	1	5708.4	Full-siblings	
	P198A- **P198C**	0.282	UR	NC	0.31	Unrelated	
	P198A- F198.1	0.270	UR	NA	0.1	Unrelated	
**12**	**P198C-** F198.1	0.731	PO	1	10637.77	Parent-offspring	CBP
	P198A- F198.3	0.315	HS	NA	0.24	Unrelated	
	**P198C-** F198.3	0.292	HS	NA	0.5	Unrelated	
	F198.1- F198.3	0.095	UR	NA	0.41	Unrelated	
	P204A- **P204C**	-0.011	UR	NC	0.03	Unrelated	
**13**	**P204A-** F204.1	0.415	PO	1	9.93	Parent-offspring	
	P204C- F204.1	0.424	PO	1	7.56	Parent-offspring	
	**P204A-** F204.2	-0.142	UR	NA	0.004	Unrelated	EPP/CBP
	P204C- F204.2	0.194	UR	NA	0.13	Unrelated	
	F204.1- F204.2	0.142	UR	NA	0.02	Unrelated	
	**P204A-** F204.3	-0.391	UR	NA	0.002	Unrelated	
	P204C- F204.3	-0.041	UR	NA	0.03	Unrelated	
	F204.1- F204.3	0.179	UR	NA	0.07	Unrelated	
	F204.2- F204.3	0.431	HS	NA	4.68	Half-siblings	
	P211A- **P211D**	-0.017	HS	NC	0.53	Unrelated	
	P211A- F211.1	0.339	PO	1	218.99	Parent-offspring	
	**P211D-** F211.1	0.121	PO	0.04	23.64	Parent-offspring	
**14**	*P211A*- F211.2	0.589	PO	1	942.79	Parent-offspring	
	**P211D-** F211.2	0.212	PO	1	3.76	Parent-offspring	
	F211.1- F211.2	0.077	HS	0.059 HS	1.51	Half-siblings	
	P211A- F211.3	0.384	PO	1	3.66	Parent-offspring	EPP/CBP
	**P211D-** F211.3	0.411	PO	1	17.84	Parent-offspring	
	F211.1- F211.3	0.095	UR	0.029 HS	0.52	Half-siblings	
	F211.2- F211.3	0.626	FS	0.827 HS	670.39	Full-siblings	

	P211A- F211.4	0.104	HS	1	0.51	Half-siblings	
	**P211D-** F211.4	0.440	PO	1	12.61	Parent-offspring	
	F211.1- F211.4	0.363	HS	0.039 HS	1.46	Half-siblings	
	F211.2- F211.4	0.107	HS	0.93 HS	2.22	Half-siblings	
	F211.3- F211.4	0.367	FS	1	43.51	Full-siblings	
	P212A- **P212B**	0.218	UR	NC	0.1	Unrelated	
**15**	*P212A*- F212.1	0.428	PO	1	4.12	Parent-offspring	
	**P212B-** F212.1	0.419	HS	1	3.66	Parent-offspring	
	P212A- F212.2	0.478	PO	1	6.55	Parent-offspring	EPP
	**P212B-** F212.2	0.460	HS	1	14.46	Parent-offspring	
	F212.1- F212.2	0.795	FS	1	2747.7	Full-siblings	
	P212A- F212.3	0.610	PO	1	55.45	Parent-offspring	
	**P212B-** F212.3	0.575	PO	1	8.11	Parent-offspring	
	F212.1- F212.3	0.267	HS	1	4.95	Half-siblings	
	F212.2- F212.3	0.320	HS	1	8.12	Half-siblings	
	**P217A-** *P217C*	0.145	FS	NC	3.73	Full-siblings	
**16**	**P217A-** F217.1	0.474	PO	NA	8.49	-	
	P217C- F217.1	0.461	PO	NA	26.7	-	
	**P217A-** F217.2	0.226	HS	NA	1.13	-	
	P217C- F217.2	0.279	HS	NA	1.97	Unrelated	
	F217.1- F217.2	0.074	HS	NA	1.07	-	
	**P217A-** F217.3	0.250	HS	NA	0.21	Unrelated	Not inferred
	P217C- F217.3	0.187	UR	NA	0.34	Unrelated	
	F217.1- F217.3	0.100	UR	NA	0.6	Half-siblings	
	F217.2- F217.3	0.648	PO	NA	70.96	-	
	**P217A-** F217.4	0.077	UR	NA	0.03	Unrelated	
	P217C- F217.4	0.208	HS	0.004	1.18	Unrelated	
	F217.1- F217.4	0.199	UR	NA	0.59	Half-siblings	
	F217.2- F217.4	0.309	UR	NA	0.55	Unrelated	
	F217.3- F217.4	0.244	UR	NA	0.58	Unrelated	
	P220A- **P220C**	0.043	HS	NC	0.13	Unrelated	
**17**	*P220A*- F220.2	0.511	PO	1	816.81	Parent-offspring	
	**P220C-** F220.2	0.393	PO	NA	6.86	Parent-offspring	
	P220A- F220.3	-0.081	HS	NA	0.42	Unrelated	

	**P220C-** F220.3	-0.022	UR	NA	0.02	Unrelated	EPP/CBP
	F220.2- F220.3	-0.144	UR	NA	0.1	Unrelated	
	P220A- F220.4	0.351	HS	NA	0.84	Unrelated	
	**P220C-** F220.4	0.407	PO	NA	0.02	Unrelated	
	F220.2- F220.4	0.175	UR	NA	0.08	Unrelated	
	F220.3- F220.4	0.132	HS	NA	1.07	Half-siblings	
	P226A- **P226C**	0.177	HS	NC	1.49	Half-siblings	
	P226A- F226.1	0.511	PO	1	194.68	Parent-offspring	
	**P226C-** F226.1	0.408	PO	NA	2.96	Unrelated	
**18**	*P226A*- F226.2	0.341	HS	0.007	5.93	Unrelated	
	**P226C-** F226.2	-0.001	UR	NA	0.01	Unrelated	EPP/CBP
	F226.1- F226.2	0.035	HS	0.972 HS	2.8	Half-siblings	
	P226A- F226.3	0.562	PO	1	188.63	Parent-offspring	
	**P226C-** F226.3	0.291	HS	NA	0.61	Unrelated	
	F226.1- F226.3	0.512	FS	0.013 HS	50.61	Half-siblings	
	F226.2- F226.3	0.475	FS	0.013 HS	78.78	Half-siblings	
	P234A- **P234D**	0.039	UR	NC	0.01	Unrelated	
	P234A- F234.1	0.450	PO	1	31.35	Parent-offspring	
**19**	**P234D-** F234.1	0.224	HS	NA	0.41	Half-siblings	
	P234A- F234.2	0.396	PO	1	47.69	Parent-offspring	
	**P234D-** F234.2	0.149	UR	NA	0.21	Unrelated	EPP/CBP
	F234.1- F234.2	0.420	HS	1 HS	8.21	Half-siblings	
	P234A- F234.3	-0.128	HS	NA	0.83	Unrelated	
	**P234D-** F234.3	0.670	PO	1	10.31	Parent-offspring	
	F234.1- F234.3	-0.177	HS	1	2.12	Half-siblings	
	F234.2- F234.3	0.307	FS	1	2.61	Half-siblings	
	**P239A-** *P239D*	-0.148	UR	NC	0.01	Unrelated	
**20**	**P239A-** F239.1	0.384	PO	1	20.1	Parent-offspring	
	P239D- F239.1	0.491	PO	1	132.35	Parent-offspring	
	**P239A-** F239.2	0.402	PO	1	11.06	Parent-offspring	
	P239D- F239.2	0.587	PO	1	52.35	Parent-offspring	Genetic monogamy
	F239.1- F239.2	0.588	FS	1	80.39	Full-siblings	
	**P239A-** F239.3	0.384	PO	1	6.27	Parent-offspring	
	P239D- F239.3	0.556	PO	1	42.7	Parent-offspring	

	F239.1- F239.3	0.558	FS	1	114.2	Full-siblings	
	F239.2- F239.3	0.782	FS	1	1474.59	Full-siblings	
	**P241.a-** *P241.b*	0.048	UR	NC	0.01	Unrelated	
**21**	**P241.a-** F241.1	0.534	PO	1	26.71	Parent-offspring	
	P241.b- F241.1	0.573	PO	1	35.28	Parent-offspring	
	**P241.a-** F241.2	0.650	PO	1	9.49	Parent-offspring	
	P241.b- F241.2	0.555	PO	1	12.55	Parent-offspring	
	F241.1- F241.2	0.636	FS	0.99	37.53	Full-siblings	
	**P241.a-** F241.3	0.306	HS	1	3.62	Parent-offspring	EPP
	P241.b- F241.3	0.312	HS	1	3.09	Parent-offspring	
	F241.1- F241.3	0.155	FS	0.99	1.44	Full-siblings	
	F241.2- F241.3	0.346	UR	NA	1.23	Half-siblings	
	**P241.a-** F241.4	0.457	PO	1	8.26	Parent-offspring	
	P241.b- F241.4	0.489	PO	1	3.91	Parent-offspring	
	F241.1- F241.4	0.245	HS	NA	1.26	Half-siblings	
	F241.2- F241.4	0.410	HS	0.99	1.06	Half-siblings	
	F241.3- F241.4	0.580	FS	0.99	378.21	Full-siblings	
	P244A- **P244C**	-0.172	UR	NC	0	Unrelated	
**22**	*P244A*- F244.1	0.223	PO	1	1.49	Parent-offspring	
	**P244C-** F244.1	0.631	PO	1	50.15	Parent-offspring	
	P244A- F244.2	0.416	PO	1	38.63	Parent-offspring	
	**P244C-** F244.2	0.442	PO	1	119.52	Parent-offspring	Genetic monogamy
	F244.1- F244.2	0.502	FS	1	17.25	Full-siblings	
	P244A- F244.3	0.401	PO	1	333.96	Parent-offspring	
	**P244C-** F244.3	0.457	PO	1	2.78	Parent-offspring	
	F244.1- F244.3	0.300	FS	1	0.97	Full-siblings	
	F244.2- F244.3	0.491	FS	1	371.11	Full-siblings	
**23**	*P250A*- **P250B**	0.031	UR	NC	0.01	Unrelated	
	P250A- F250.1	0.512	PO	1	29.3	Parent-offspring	
	**P250B-** F250.1	0.485	PO	1	13.24	Parent-offspring	Genetic monogamy
	P250A- F250.2	0.330	PO	1	6.94	Parent-offspring	
	**P250B-** F250.2	0.712	PO	1	237.02	Parent-offspring	
	F250.1- F250.2	0.792	FS	1	1699.45	Full-siblings	

	**P252A-** *P252D*	0.189	UR	NC	0.24	Unrelated	
**24**	**P252A-** F252.1	0.590	PO	1	27.95	Parent-offspring	
	P252D- F252.1	0.619	PO	1	183.79	Parent-offspring	
	**P252A-** F252.2	0.424	PO	1	41.24	Parent-offspring	
	P252D- F252.2	0.723	PO	1	269.77	Parent-offspring	Genetic monogamy
	F252.1- F252.2	0.621	FS	1	556.22	Full-siblings	
	**P252A-** F252.3	0.666	PO	1	1276.27	Parent-offspring	
	P252D- F252.3	0.533	PO	1	4.18	Parent-offspring	
	F252.1- F252.3	0.650	FS	1	17.65	Full-siblings	
	F252.2- F252.3	0.479	FS	1	109.03	Full-siblings	
	**P253.a-** *P253.c*	0.340	HS	NC	6.6	Half-siblings	
**25**	**P253.a-** F253.1	0.584	PO	1	925.66	Parent-offspring	
	P253.c- F253.1	0.594	PO	1	368.39	Parent-offspring	
	**P253.a-** F253.2	-0.089	UR	NA	0.03	Unrelated	
	P253.c- F253.2	-0.208	UR	NA	0	Unrelated	
	F253.1- F253.2	-0.032	UR	NA	0.01	Unrelated	
	**P253.a-** F253.3	0.566	PO	1	802.06	Parent-offspring	
	P253.c- F253.3	0.632	PO	1	3380.28	Parent-offspring	
	F253.1- F253.3	0.358	HS	0.99	17.18	Half-siblings	EPP/CBP
	F253.2- F253.3	-0.021	HS	1	0.23	Half-siblings	
	**P253.a-** F253.5	0.228	PO	0.65	3.07	Parent-offspring	
	P253.c- F253.5	0.177	UR	NA	0.69	Unrelated	
	F253.1- F253.5	0.378	HS	0.65	7.2	Half-siblings	
	F253.2- F253.5	-0.043	UR	NA	0.26	Half-siblings	
	F253.3- F253.5	0.299	HS	0.65	7.86	Half-siblings	
	**P254.a-** *P254.b*	0.294	HS	NC	2.04	Unrelated	
**26**	**P254.a-** F254.1	0.704	PO	1	11222.63	Parent-offspring	
	P254.b- F254.1	0.663	PO	1	1325.91	Parent-offspring	
	**P254.a-** F254.2	0.468	PO	1	68.95	Parent-offspring	
	P254.b- F254.2	0.441	PO	1	21.91	Parent-offspring	Genetic monogamy
	F254.1- F254.2	0.544	FS	0.99	779.2	Full-siblings	
	**P254.a-** F254.3	0.660	PO	1	3253.64	Parent-offspring	
	P254.b- F254.3	0.619	PO	1	240.3	Parent-offspring	
	F254.1- F254.3	0.835	FS	0.99	885242.85	Full-siblings	

	F254.2- F254.3	0.431	HS	0.99	244.44	Full-siblings	
	P256.a- **P256.b**	-0.006	UR	NC	0.04	Unrelated	
**27**	*P256.a*- F256.1	0.756	PO	1	7683.62	Parent-offspring	
	**P256.b-** F256.1	0.265	PO	1	7.77	Parent-offspring	
	P256.a- F256.2	0.395	PO	1	156.32	Parent-offspring	
	**P256.b-** F256.2	0.125	PO	1	21.6	Parent-offspring	Genetic monogamy
	F256.1- F256.2	0.352	FS	1	175.92	Full-siblings	
	P256.a- F256.3	0.394	PO	1	15.61	Parent-offspring	
	**P256.b-** F256.3	0.531	PO	1	378.19	Parent-offspring	
	F256.1- F256.3	0.280	HS	1	13.47	Full-siblings	
	F256.2- F256.3	0.419	FS	1	8124.25	Full-siblings	
	P76B- **P76D**	0.288	FS	NC	1.69	Half-siblings	
**28**	*P76B*- F76.1	0.474	PO	1	19.83	Parent-offspring	
	**P76D-** F76.1	0.413	PO	0.85	6.21	Parent-offspring	Genetic monogamy
	P76B- F76.2	0.474	PO	1	19.83	Parent-offspring	
	**P76D-** F76.2	0.413	PO	0.85	6.21	Parent-offspring	
	F76.1- F76.2	1.000	FS	1	24593.25	Full-siblings	
	P199.a- **P199.b**	-0.162	UR	NC	0	Unrelated	
**29**	*P199.a*- F199.1	-0.244	UR	NA	0	Unrelated	
	**P199.b-** F199.1	0.275	HS	NA	0.21	Half-siblings	
	P199.a- F199.3	0.406	PO	1	125	Parent-offspring	EPP/CBP
	**P199.b-** F199.3	0.431	PO	NA	1.14	Unrelated	
	F199.1- F199.3	0.072	UR	NA	0.04	Unrelated	
	P199.a- F199.4	-0.185	HS	NA	0.22	Half-siblings	
	**P199.b-** F199.4	0.362	HS	NA	1.18	Unrelated	
	F199.1- F199.4	-0.047	UR	NA	0	Unrelated	
	F199.3- F199.4	0.388	HS	0.853	142.35	Half-siblings	
**30**	*P74A*- **P74B**	0.307	HS	NC	0.23	Unrelated	
	P74A- F74.1	0.550	PO	1	21.49	Parent-offspring	
	**P74B-** F74.1	0.208	HS	NA	0.03	Unrelated	EPP
	P74A- F74.4	0.626	PO	0.073	38.61	Parent-offspring	
	**P74B-** F74.4	0.065	UR	NA	0.04	Unrelated	
	F74.1- F74.4	0.473	FS	1	18.99	Full-siblings	

**31**	**P209A-** *P209C*	-0.161	UR	NC	0.87	Unrelated	
	**P209A-** F209.1	0.360	PO	1	1565.61	Parent-offspring	
	P209C- F209.1	0.296	FS	1	8.19	Parent-offspring	Genetic monogamy
	**P209A-** F209.2	0.553	PO	1	2837.69	Parent-offspring	
	P209C- F209.2	0.229	PO	1	782.44	parent-offspring	
	F209.1- F209.2	0.354	FS	1	1281.37	Full-siblings	
